# Fluorescent Metal–Organic Framework Nanoparticles for Explosive Detection

**DOI:** 10.1021/acs.jpcc.5c00753

**Published:** 2025-06-05

**Authors:** Raymond Yu, Tiffany Nguyen, Victor H. Cortez, Boyang Chen, Kristi M. Ishihara, Enrico Tapavicza, Fangyuan Tian

**Affiliations:** † Department of Chemistry and Biochemistry, 209686 California State University Long Beach, Long Beach, California 90840, United States; ‡ Faculty of Chemistry and Pharmacy, Institute of Physical and Theoretical Chemistry, University of Regensburg, 93040 Regensburg, Germany

## Abstract

Fluorescent metal–organic framework (MOF) nanoparticles were prepared by doping zeolitic imidazolate framework-8 (ZIF-8) with fluorescein through ″one-pot″ synthesis. The resulting fluorescein@ZIF-8 (F@ZIF-8) material exhibits a luminescent “turn-off” response toward nitroaromatic explosives. We performed a combined experimental and computational study to understand the structural properties and formation mechanisms of the prepared F@ZIF-8 composite and to explain the solid-state fluorescence and quenching in the presence of a nitroaromatic analyte. We confirmed that fluorescein can be encapsulated within the ZIF-8 cages through electrostatic interactions between dianionic fluorescein and Zn open metal sites. The scaffold of ZIF-8 can reduce the aggregation-caused quenching (ACQ) effect of fluorescein and increase the quantum yield to 69.7 ± 0.1%. The photophysical mechanism explains the fluorescence emission of F@ZIF-8 in the solid state and the fluorescence quenching in the presence of nitroaromatic analytes, including 2,4,6-trinitrophenol (TNP), trinitrotoluene (TNT), 4-nitrotoluene, nitrobenzene, and 2-nitropropane. Our material exhibits a high sensitivity toward TNP with a limit of detection (LOD) of 2 μM. This work presents a new opportunity for designing luminescent porous materials for “signal-off” sensing and demonstrates the potential of using luminescent MOFs as an analytical tool for explosive detections.

## Graphical abstract

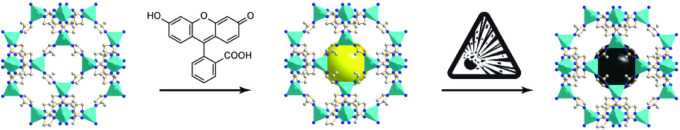

## Introduction

Explosive devices are one of the most common forms of terrorism, accounting for many historical terrorist attacks. The explosive threats are projected to pose heightened risks in war zones due to increased activities by terrorist groups including suicide bombers and armed insurgents. According to the unclassified Global Terrorism Database from the U.S. Department of Homeland Security, 48.9% of the terrorist attacks have been related to bombings since 1970.^1^ Moreover, undetonated ordnance presents a persistent detonation hazard and contributes to long-term human health risks and environmental contamination. Based on molecular structures, usages, and raw material accessibilities, common explosives can be classified into six categories:^2,3^ nitroalkanes and nitroaromatic compounds (NACs), such as 2,4,6-trinitrotoluene (TNT), 1,3,5-trinitrobenzene (TNB), and nitromethane (NM); nitramines and nitrate esters, for example, hexahydro-1,3,5-trinitro-1,3,5-triazine (RDX) and octahydro-1,3,5,7-tetranitro-1,3,5,7-tetrazocine (HMX), also known as plastic explosives; ammonium salts, commonly used in mining and blasting, widely available due to their use as agricultural fertilizers; and finally, peroxide-based explosives, including hydrogen peroxide, triacetone triperoxide (TATP, acetone peroxide), and other peroxides, which are commonly used in improvised explosive devices due to their easy accessibility and synthesis.^4^ In this work, we focus on the detection of NACs since they are most commonly used in the military and landmines.^5^ According to the United Nations Mine Action Service (UNMAS), accidental detonation of these hidden landmines still results in the death or injury of many people a year with an 80% civilian casualty rate.^6^ Even if left undetonated, these hidden ordnances constitute a significant environmental risk due to chemical leaching and potential groundwater contamination.^7,8^ In this work, we studied the detection of five explosives, including 2,4,6-trinitrophenol (TNP), trinitrotoluene (TNT), 4-nitrotoluene (NT), 2-nitropropane (NP), and 1,3,5-trinitro-1,3,5-triazinane (RDX), and an NAC explosive simulant, nitrobenzene (NB).

Current available explosive detection techniques include trained detection canines, ion mobility spectrometry (IMS), X-ray machines, fluorescent polymers, and field-effect transistors.^9,10^ Sniffing dogs are trained to detect the volatile organic compounds (VOCs) emitted by certain explosives; however, they exhibit low sensitivity to plastic explosives, which possess lower volatility than NACs and emit negligible VOCs. Moreover, trained canines may experience olfactory fatigue and reduced performance due to environmental interferences and competing odorants.^11^ IMS and other mass spectrometry-based techniques, such as gas chromatography coupled mass spectrometry (GC-MS), are used in airports and laboratories for explosive detection by monitoring the mass-to-charge ratio (*m*/*z*) of nitro ions with high sensitivity.^10^ However, these bulky MS instruments are not suitable for field detection. Therefore, fast-response optical methods have emerged as cost-effective and portable alternatives. These optical methods track variations in the absorbance or fluorescence intensity of probe molecules in response to explosive analytes. Fluorescence-based optical detection for explosives has been developed in the last two decades. The detection systems have evolved into supramolecular clusters and conjugated polymers with amplified fluorescence responses.^3,12,13^ However, the relatively low surface area of these fluorescent materials constrains their dynamic detection range. Therefore, luminescent metal–organic frameworks (MOFs), which have extremely high surface areas, represent promising candidates for future explosive detection applications.^14–16^

MOFs are a class of hybrid materials consisting of transition-metal ions/clusters coordinated to organic linkers forming periodic framework architectures.^17^ Due to their adaptable structures and exceptionally high surface areas, MOFs have been extensively investigated for use in gas storage and separation, catalysis, energy conversion, and guest molecule encapsulation.^18–23^ Compared to conventional mesoporous and microporous materials, such as zeolites, carbon molecular sieves, and porous metal oxides,^24,25^ MOFs demonstrate several structural and functional advantages over conventional porous materials: first, the tunable porosity arises from the coordination geometry of metal nodes and organic building blocks, facilitating systematic structural design.^26^ Second, the organic ligands in MOFs are feasible for postsynthetic modification (PSM), allowing for the incorporation of additional functionalities.^27^ Third, the large porosity range makes MOFs suitable for holding guest molecules or nanoparticles in different sizes with various features, rendering MOFs an effective scaffold for catalytic applications.^21^ Moreover, MOFs synthesized under versatile conditions have been employed in the development of luminescent devices due to their tunable optical and electronic properties.^28^

Selective MOFs exhibit a range of luminescent behaviors, including fluorescence, phosphorescence, and scintillation.^29^ Due to the hybrid nature of MOFs, luminescence can originate from organic linkers,^30^ metal clusters,^31^ adsorbed luminescent guest molecules or ions,^32,33^ or due to MOF–adsorbate interactions or interactions with surrounding solvent molecules.^34–36^ Compared to other fluorescent materials, MOFs offer modular structural tunability, selective adsorption capacities, multifunctional structures, and mechanical flexibility.^37–39^ In addition, MOFs possessing high pore volume can offer accessible internal and external binding sites for fluorophores and analyte molecules.^40^ Recent research has focused on employing luminescent MOFs (LMOFs) in chemical sensing, optoelectronics, and bioimaging applications.^41–43^

This work investigates the optical properties of a representative LMOF, fluorescein-encapsulated ZIF-8 (F@ZIF-8), toward nitroaromatic explosives. ZIF-8 is composed of zinc ions connected with 2-methylimidazole linkers by forming a sodalite (SOD) crystal structure.^44^ Compared with other MOFs, ZIF-8 was selected as the host material for loading fluorescein, primarily due to the following reasons: first, the pore geometry of ZIF-8 is well suited for encapsulating fluorescein. ZIF-8 features a pore aperture of 3.4 Å and an inner cage diameter of 11.6 Å,^45^ while fluorescein has a molecular size of approximately 2 × 7 Å.^46^ These dimensions suggest that fluorescein can be effectively trapped within the ZIF-8 cages, where their intramolecular motion can be restricted. This confinement can suppress raditionless decay, thereby enhancing the fluorescence efficiency of fluorescein. Second, ZIF-8 can be synthesized under mild room-temperature conditions, which minimizes the risk of degradation of fluorescein. This is particularly advantageous because other MOFs with similar pore structures typically require solvothermal or hydrothermal conditions for synthesis,^45^ which could significantly reduce the quantum yield (QY) of fluorescein.^47^ Therefore, ZIF-8 was chosen as the host material for fluorescein encapsulation and investigated for its potential in explosive sensing applications. Based on previously reported studies on fluorescence-based explosive detection, sensing mechanisms are generally categorized as follows: photoinduced electron transfer (PET), fluorescence resonance energy transfer (FRET), and diffusional exchange energy transfer.^2,48^ Most explosive NACs contain electron-withdrawing nitro moieties with electron deficiency on nitrogen atoms. In contrast, fluorophores are electron-rich compounds capable of donating electrons to electron-deficient nitro groups via photoinduced electron transfer. During these donor–acceptor interactions, variations in fluorescence intensity (primarily quenching), emission wavelength, or excited-state lifetime serve as indicators of NAC detection. To date, the majority of studies have concentrated on the synthesis of LMOFs. In comparison, fewer studies have addressed the interaction mechanisms of the formed LMOFs. Fluorescein and its derivatives have been used as fluorescent probes due to their high QY and pH-dependent behaviors.^49^ As a prototypical member of the xanthene family of organic dyes, fluorescein’s spectroscopic and photochemical properties depend on the electronic structure of the planar-conjugated core heterocycle. Fluorescein is functionalized at the 9-position with an o-benzoate moiety, which enables tautomerization among quinoid, lactoid, and zwitterionic states, as shown in Scheme 1.^50^ The crystal structures of these three tautomers were reported to have different colors.^50^ The most common tautomer in the solid is quinone, which is dark red (Figure 1a, left vial). The ionization of fluorescein is pH-dependent (Scheme 1). Only the monoanion and dianion forms of fluorescein are fluorescent.^51^ This fluorescence property is exploited for the detection of selective NAC explosives in the present study.

**Scheme 1:**
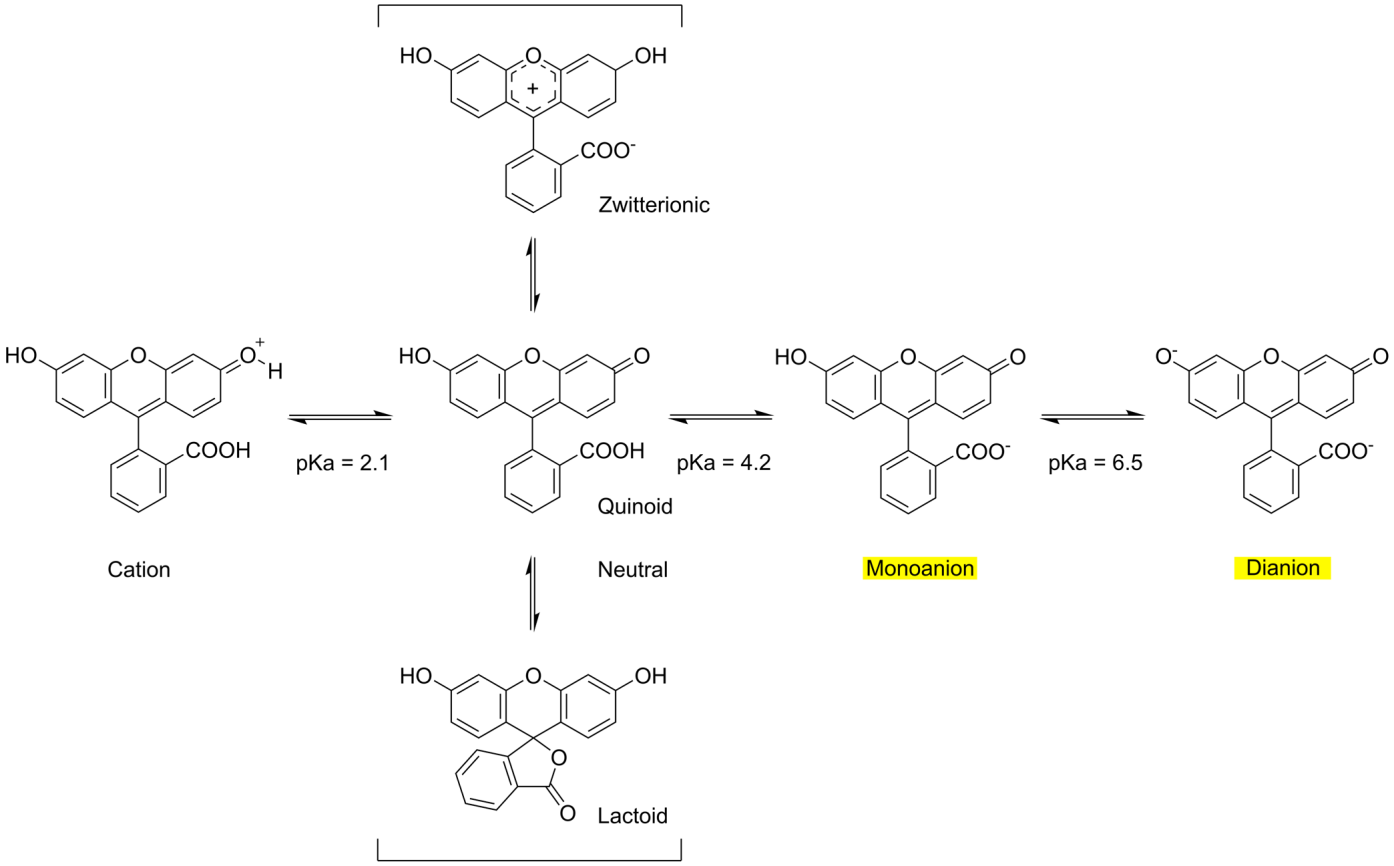
Different Ionic and Neutral Forms of Fluorescein^a^

a The tautomers of fluorescein are circled in the box. Only monoanion and dianion fluorescein can emit fluorescence as indicated in a highlight color. Figure adapted from ref.^51^

**Figure 1:**
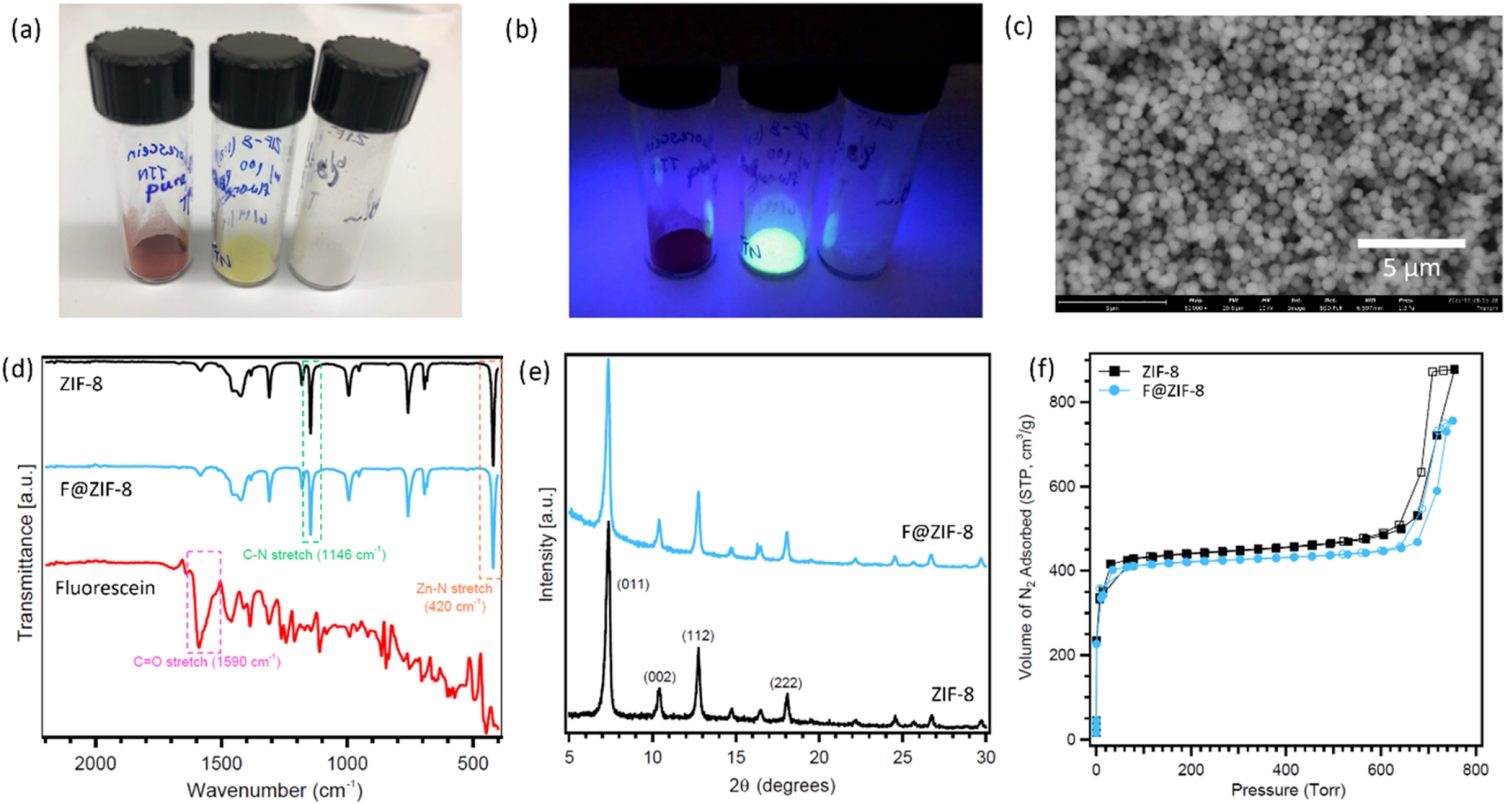
(a) and (b) Optical images of solid fluorescein dye (red powder, left), F@ZIF-8 (yellow powder, middle), and pristine ZIF-8 (white powder, right) under visible (a) and UV (b) conditions; (c) SEM image of F@ZIF-8; (d) ATR-IR spectra of pristine ZIF-8 (black), F@ZIF-8 (blue), and solid fluorescein (red); (e) PXRD patterns of pristine ZIF-8 (black) and F@ZIF-8 (blue); and (f) N_2_ sorption isotherms of pristine ZIF-8 (black) and F@ZIF-8 (blue) at 77 K in the pressure range of 0–800 Torr. Solid symbols indicate adsorption curves, and open symbols indicate desorption curves.

Kwon, Lah, and co-workers reported that photoinduced electron transfer and resonance energy transfer are the primary quenching mechanisms of a type of OH-functionalized MOF in water, while exciton migration is responsible for fluorescence quenching of the same MOF in chloroform.^52^ Tan and co-workers reported the incorporation of fluorescein as a guest molecule in ZIF-8 nanoparticles with a QY of approximately 98%, which was subsequently applied in solid-state lighting.^53^ The same group also reported a dual-guest system by encapsulating both fluorescein and rhodamine B in ZIF-8, which exhibited a bright yellow emission under UV light and was integrated into a 3D printable white light-emitting device.^54^ In this work, fluorescent F@ZIF-8 was synthesized via a “one-pot” approach. We investigated the host–guest interactions between fluorescein and ZIF-8 by combining experimental and computational approaches. Additionally, we assessed the performance of F@ZIF-8 in detecting NAC explosives.

## Experimental and Computational Methods

### Chemicals and Materials

All chemicals and reagents were obtained from commercial sources and used without further purification. Details are listed in the SI.

### Synthesis and Characterization of ZIF-8 and Fluorescein@ZIF-8

Pristine nanoscale ZIF-8 was synthesized based on the previously reported literature^55^ with minor modifications. ZIF-8 was prepared by mixing a 1:1 v/v ratio of precursor solutions of 50 mM zinc nitrate hexahydrate (Zn­(NO_3_)_2_·6H_2_O) and 0.4 M 2-methylimidazole (2-mIm) in methanol and stirring for 1 h at room temperature. The resulting mixture was centrifuged at 7500 rpm for 30 min. Afterward, the precipitate was resuspended in anhydrous ethanol and centrifuged to remove the exchanged solvent. The suspension-centrifugation cycle was repeated twice to completely remove excess reagent. The resulting precipitate was dried in a vacuum oven at 90 °C overnight to afford white powder as pristine ZIF-8 (Figure 1a, right vial).

Synthesis of F@ZIF-8 is identical to that of pristine ZIF-8, except a desired amount of fluorescein was dissolved in the 2-mIm precursor solution and then mixed with the Zn^2+^ solution. The equivalents of fluorescein were calculated as a molar percentage, with respect to Zn^2+^. The molar ratios for Zn^2+^:2-mIm:fluorescein are 1:8:X, where X varies from 0.3 to 30 mol %. Since fluorescein was added during the ZIF-8 formation, we called this method a “one-pot” synthesis.

### Fourier Transform Infrared Spectroscopy

Vibrational spectra of ZIF-8 and F@ZIF-8 powders were acquired on a Bruker α I Fourier transform infrared spectrometer (FTIR) equipped with a diamond-attenuated total reflectance (ATR) accessory. All IR spectra were collected in the range of 450–4000 cm^–1^ at an 8 cm^–1^ resolution and a total of 64 scans per spectrum. Ambient air was used as the background.

### Powder X-ray Diffraction

Powder X-ray diffraction (PXRD) patterns of ZIF-8 and F@ZIF-8 were performed on a Bruker D2 Phaser diffractometer with a Cu Kα1 X-ray source (λ=1.5406 Å) at 30 kV in a range of 2theta from 5° to 45° with a step size of 0.02° on either a PMMA holder or a Si low background sample holder.

### Nitrogen Sorption Analysis

A Micromeritics ASAP 2020 Plus was used to measure the BET surface area and pore volume of ZIF-8 and F@ZIF-8 samples using N_2_ sorption isotherms at 77 K with gas pressure up to 800 Torr. Prior to each measurement, approximately 100 mg of sample was degassed through a multistep heating process from room temperature to 150 °C and degassed at 150 °C overnight. During N_2_ sorption isotherm measurements, the sample tube was kept in a liquid N_2_ bath to maintain the temperature at 77 K. Ultra-high-purity grade N_2_ (99.99%) was used for all N_2_ isotherm measurements.

### Scanning Electron Microscopy

The morphology of the formed F@ZIF-8 nanoparticles was examined using a Phenom ProX G6 scanning electron microscope (SEM) equipped with energy-dispersive X-ray spectroscopy (EDS) analysis. The SEM images were taken with an acceleration voltage of 10 kV using a backscatter detector (BSD) with a working distance of around 6.6 mm in vacuum conditions. The acceleration voltage was adjusted to 15 kV for the EDS analysis. All samples were mounted on a charge reduction sample holder for imaging.

### Fluorescence Quantum Yield Measurement

Fluorescence QYs of F@ZIF-8 with various fluorescein doping amounts were measured using a PL quantum yield system (C11347-11, Hamamatsu photonics). The excitation wavelength range was 400–800 nm with a 25 nm step. Pristine ZIF-8 in air was used for the reference reading. All F@ZIF-8 samples were dried in a vacuum oven at 90 °C for 24 h prior to QY measurements. A quartz dish was used as the sample holder.

### Detection of Explosives

Six NAC explosives or explosive simulants were tested as quenchers. Unless otherwise stated, fluorescence measurements were conducted using a solution of 1 mg of F@ZIF-8 to 2.5 mL of ACN (equivalent to 0.4 mg/mL). The solution was sonicated until all large particles were dispersed into a cloudy suspension and tested on the same day. All spectra were collected using a Shimadzu RF-6000 with an excitation wavelength of 490 nm and a scan range of 500–700 nm. The fluorescence quenching signals were compared at an emission wavelength of 515 nm. For quenching experiments, all tested explosive analytes along with their concentrations are summarized in Table S1. A quartz cuvette was filled with 2.5 mL of a F@ZIF-8 solution and set to stir in the fluorometer at room temperature. After an initial reading, 20 μL of analyte was added, and an emission spectrum was collected. This process was repeated until a total of 200 μL of analyte was added. Quenching efficiency was evaluated for each explosive analyte using the Stern–Volmer equation

1 $$I_{0}/I=K_{\mathrm{sv}}\left[Q\right]+1$$

where *I*_0_ is the fluorescence emission intensity before adding the quencher (analyte), *I* is the emission intensity in the presence of the quencher, [*Q*] is the molar concentration of the quencher, and *K*_sv_ is known as the Stern–Volmer constant or quenching constant (M^–1^). Once a linear range of quencher concentrations was identified, the limit of detection (LOD) was calculated based on eq 2

2 $$\mathrm{LOD}=\frac{3\sigma }{S}$$

where σ is the standard deviation of the blank measurement without the quencher, and *S* is the slope of the linear calibration curve of ΔI, the difference between *I*_0_ and *I*, as a function of [*Q*].

### Computational Details

Electronic structure calculations of a reduced model of F@ZIF-8, containing a [Zn­(mIm)_3_]^−1^ cluster plus one fluorescein dianion molecule, were performed with the Gaussian 16 suite of programs (Rev. A.03)^56^ using the B3LYP approximation to the exchange–correlation functional^57–59^ with the LANL2DZ basis set.^60,61^ Ground-state equilibrium structures were optimized using default convergence criteria, as provided by Gaussian. Vibrational spectra were calculated to confirm that energetic minima were reached. To assess the complexation energy between [Zn­(mIm)_3_]^−1^ and the fluorescein dianion, we computed the interaction energy using counterpoise correction, both with and without the Grimme D3-dispersion correction.^62^ We assessed two different structures, one in which the carboxylate group coordinates to the Zn^2+^ ion (FlCOO-ZIF-8) and one in which the phenoxylate group coordinates to the Zn^2+^ ion in ZIF-8 (FlO-ZIF-8).

Excited-state calculations using time-dependent density functional theory (TDDFT) and second-order approximate coupled cluster theory (CC2)^63^ were carried out using Turbomole 7.4.^64^ In both cases, we used ground-state equilibrium structures optimized using B3LYP/LANL2DZ plus D3-correction. TDDFT/B3LYP excitation energies for the five lowest excited states were computed using the TZVP basis set.^65^ The conductor-like screening model (COSMO)^66^ was used to mimic the solvent (ethanol). We used a dielectric constant of 24.3 and a refractive index of 1.36. CC2 calculations were carried out using the SVP basis set^65^ and the resolution of identity approximation and the frozen core approximation,^63^ as provided by default by Turbomole. Since CC2 calculations using COSMO did not result in stable convergence, we only report results of the gas-phase calculations.

## Results and Discussion

### Interactions between Fluorescein and ZIF-8 in F@ZIF-8

In our initial study, the reaction system of ZIF-8 (5 mmol) was mixed with fluorescein (5 mg, equivalent to 0.3 mol %) in a “one-pot” synthesis condition. The resulting F@ZIF-8 exhibits a pale-yellow color as compared to a white powder of pristine ZIF-8 and a dark red color of fluorescein in quinone form (Figure 1). Interestingly, when these materials are illuminated by a hand-held UV lamp (λ = 365 nm), only the composite F@ZIF-8 emits fluorescence (Figure 1b). Spontaneous emission is completely quenched in a red fluorescein powder due to the parallel orientation of tightly stacked hydroxy-xanthone planes in the crystalline phase. The face-to-face π–π interactions in the aggregated state are also responsible for the quenching effects in concentrated solutions of fluorescein.^67^ The observed fluorescence of the formed solid-state F@ZIF-8 could be attributable to the dispersion of monoanionic or dianionic fluorescein within ZIF-8. Considering the pH of the ZIF-8 synthesis solution (pH 9–12 to promote the deprotonation of imidazole), the majority of fluorescein should be in its dianionic form in F@ZIF-8. The synthesized solid-state F@ZIF-8 shows an absorbance λ_max_ at 490 nm and an emission at 519 nm (Figures S2 and S3). The yellow color of F@ZIF-8 may be due to the presence of the dianionic state fluorescein or simply due to the dilution of quinone state fluorescein with ZIF-8. To confirm that, we performed a control experiment by physically mixing solid fluorescein and presynthesized pristine ZIF-8 dry powder in the same ratio as the “one-pot” synthesis condition. The mixture showed a color of pale pink and did not emit any fluorescence under the UV condition (Figure S1, left vial). We carried out two other control experiments by mixing fluorescein and ZIF-8 in a “wet” condition: fluorescein was added to the presynthesized ZIF-8 ethanol solution, and then the solid mixture was dried with and without three times of rinsing with ethanol. The details of all control experiments are provided in the SI. Both “wet-mixed” control samples exhibited an orange color (Figure S1, middle and right vials), and they both show fluorescence under the UV irradiation. We think it is due to some degree of surface adsorption of fluorescein on preformed ZIF-8 nanocrystals, and the surface-adsorbed fluorescein cannot be easily washed off by ethanol during regular centrifugation and dispersion cycles. This finding is consistent with our previous work on rhodamine B adsorption on ZIF-8;^68^ it is possible that some fluorescein molecules being adsorbed on the outer surface of preformed ZIF-8 interact with surface defects of ZIF-8 nanocrystals. There are two questions we would like to answer: (1) What are the interactions between fluorescein and ZIF-8 in F@ZIF-8 prepared by the “one-pot” synthesis? and (2) Does the loading amount of fluorescein affect the luminescence properties of F@ZIF-8?

To answer the first question, we started by studying the location of fluorescein in F@ZIF-8, which will enable a better understanding of the structure–property relationships and provide insight into the interactions between guest molecules and their host materials. There are three possible locations for fluorescein in F@ZIF-8: (1) on the surface of ZIF-8; (2) within the ZIF-8 particles; and (3) inside of ZIF-8 cages. We first examined the chemical composition and crystal structure of F@ZIF-8. The IR spectra of F@ZIF-8, pristine ZIF-8, and pure fluorescein dye were collected and are shown in Figure 1d. The vibrational frequency peaks involving molecular stretching from 2-mIm and Zn–N were consistent with those reported in the literature.^53^ The Zn–N stretching and C–N stretching peaks were observed at 420 and 1146 cm^–1^, respectively, which are the characteristic IR features of ZIF-8. More importantly, the IR spectra of ZIF-8 and F@ZIF-8 are almost identical, indicating that the addition of fluorescein to the ZIF-8 synthesis did not cause any changes in the chemical composition of ZIF-8. The results further indicated that most of the fluorescein in F@ZIF-8 was not adsorbed on the surface of ZIF-8, or at least not to a level detectable by ATR-IR. Zhang et al. reported similar observations when they characterized ZIF-8 loaded with rhodamine B and fluorescein disodium salt dyes prepared by postsynthetic surface adsorption.^69^ Further discussions will be made on the basis of the computational results below. Computational Details Figure 1e compares the XRD patterns of F@ZIF-8 and pristine ZIF-8, exhibiting a high alignment between those two and no shift of diffraction peaks, and is consistent with previously reported ZIF-8 crystal structures.^68,70^ This indicated that the addition of fluorescein to the ZIF-8 synthesis did not cause any shrinkage or swelling of the ZIF-8 crystal cells. Considering the pore aperture and cage diameter of ZIF-8 (3.4 and 11.6 Å)^44^ and the size of fluorescein (with estimated semiaxis lengths of 2 and 7 Å),^46^ fluorescein molecules are likely trapped inside the ZIF-8 cage during the “one-pot” synthesis. Xiong et al. reported that fluorescein can be encapsulated within ZIF-8 and form a guest–host system while a high QY of fluorescein remained.^53^ Our XRD results confirmed that the crystal structure of ZIF-8 was not altered by the fluorescein doping. However, the results did not provide conclusive evidence that fluorescein is located within the ZIF-8 pores. Therefore, we turned to the N_2_ sorption analysis, which measures the N_2_ adsorption and desorption isotherms on F@ZIF-8 at 77 K. If the pores of ZIF-8 are occupied by guest molecules, the N_2_ sorption amount should be noticeably decreased since less space in the framework would be available for loading N_2_ molecules. Figure 1e compares the N_2_ sorption isotherms of both F@ZIF-8 and pristine ZIF-8 at 77 K. Both materials adsorbed a large amount of N_2_ at low pressure conditions and exhibited a type I isotherm, which is consistent with previously reported isotherm data for ZIF-8.^71^ The isotherm observed on F@ZIF-8 was very close to that of pristine ZIF-8, which confirms that the microporous structure of ZIF-8 was not affected by fluorescein doping. Additionally, an H1-type hysteresis loop was observed on the isotherms of both materials when the pressure was close to atmospheric pressure. The hysteresis loop is typically attributed to thermodynamic or network effects of a porous material. An H1-type hysteresis loop indicates a relatively high pore size uniformity; it can be caused by the capillary condensation of N_2_ sorption occurred in uniform nanoparticles with facile pore connectivity.^72^ Our SEM study (Figure 1c) also confirmed that ZIF-8 retains its uniformity with fluorescein doping. The BET surface areas of ZIF-8 and F@ZIF-8 are 1697.7 and 1661.8 m^2^/g, respectively. The 2% decrease in surface area may be due to the encapsulation of a small amount of fluorescein within the ZIF-8 pores, but it can also be caused by encapsulation within ZIF-8 nanocrystals or surface adsorption. To further confirm the location of fluorescein, we performed a series of N_2_ gas sorption measurements on F@ZIF-8 with different fluorescein doping amounts. Figure 2 shows the BET N_2_ gas sorption isotherms of F@ZIF-8 measured at 77 K. All materials were degassed at an elevated temperature (150 °C) in a vacuum to remove trapped air prior to measurements. We notice that the N_2_ sorption amount slightly decreases with an increasing fluorescein doping level, and the steepness of the hysteresis loop decreases until it completely disappears at a fluorescein loading amount of 10 mol %. We also compare the BET surface areas, external surface areas, and micropore volumes of these F@ZIF-8 samples (Table S1). We observe a consistent trend that all of these parameters are inversely correlated with the fluorescein doping level, indicating that fluorescein indeed occupies ZIF-8 pores, especially when the doping amount is high. By combining our IR, XRD, and N_2_ sorption analyses, we conclude that the majority of fluorescein molecules are encapsulated inside ZIF-8 cages. Fluorescein molecules are dispersed within the ZIF-8 cages, thereby preventing self-quenching. Additionally, due to the spatial confinement of ZIF-8, the intramolecular motion of fluorescein is restricted within the cages, resulting in the formation of a fluorescent composite. Other studies demonstrated a similar approach by the encapsulation of copper nanoclusters (CuNCs) and Ag-doped Au nanoclusters (Ag/AuNCs) within ZIF-8 cages to produce highly emissive nanocrystals.^73,74^

**Figure 2:**
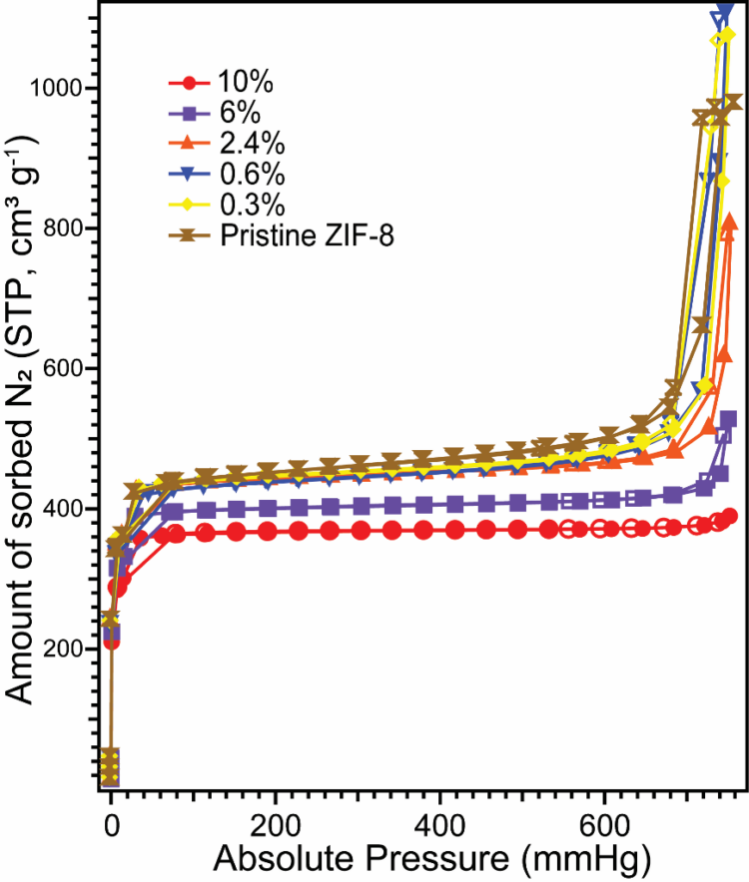
N_2_ sorption isotherms of F@ZIF-8 with variable fluorescein doping amounts. Solid symbols represent adsorption data; empty symbols represent desorption data.

Once the location of fluorescein in F@ZIF-8 is confirmed, we begin to explore the interactions between fluorescein and ZIF-8 in F@ZIF-8 by using both experimental and computational tools. We first test with a salt metathesis reaction by adding uranine (the disodium salt of fluorescein) to the Zn^2+^ precursor solution during the ZIF-8 synthesis. We notice that a red, amorphous precipitate is formed immediately. This phenomenon reveals a strong interaction between fluorescein and Zn^2+^. Since fluorescein has two possible ionizable groups, we hypothesize that fluorescein dianion might be bound through electrostatic interactions between the carboxylate or the phenoxide with the Zn^2+^ cation from the open metal sites in ZIF-8. The geometries for the two possible configurations, carboxylate-bound (FlCOO-ZIF-8) and phenoxide-bound (FlO-ZIF-8), are optimized using Gaussian at the B3LYP/LAN2LDZ level with and without the Grimme D3-dispersion correction (Figure S4). Vibrational spectra are calculated (Figure S5), and no imaginary frequencies are detected, confirming the energetic minima of the optimized structures. The counterpoise-corrected interaction energies between the fluorescein dianion and the remaining cluster formed by Zn^2+^ and three imidazole units [Zn­(mIm)_2_]^−1^ of both configurations are listed in Table S2. Without D3-correction, the complexation energies of the two structures only differ by 0.43 kcal/mol, indicating that both geometries are very likely to coexist. With the D3-correction, the complexation energies of both configurations are still similar (18.94 vs 16.95 kcal/mol), with the FlO-ZIF-8 geometry being slightly more favorable (Table S2). We conclude that fluorescein can coordinate to the open Zn site in ZIF-8 through either carboxylate and/or phenoxide groups, with a slight preference for coordination through the phenoxide group.

To further study the interactions between fluorescein and ZIF-8, we compute TDDFT excitation energies for the five lowest excited states of both structures (FlO-ZIF-8 and FlCOO-ZIF-8) on the TDDFT/B3LYP level plus implicit solvent (Table 1) and CC2/SVP in the gas phase (Table S3). Excited states obtained by CC2 are qualitatively similar to to the ones obtained by TDDFT but agree slightly better with respect to the absolute value of the main absorption band (Table S3). The good agreement between CC2 and TDDFT shows that the possible charge-transfer failure of TDDFT^75,76^ is not an issue in this system when the B3LYP approximation is used. In the following, we therefore analyze only the TDDFT-computed spectra, as these include the implicit solvent model.

**Table 1:** List of Predicted TDDFT/B3LYP Wavelengths, Oscillator Strengths, and Dominating Orbital Contributions of the Five Lowest Excited States of the Phenoxide-Bound (FlO-ZIF-8) and Carboxylate-Bound (FlCOO-ZIF-8) Configurations

	FlO-ZIF-8			FlCOO-ZIF-8		
singlet excited state #	wavelength (nm)	oscillator strength (au)	orbital contributions^a^	wavelength (nm)	oscillator strength (au)	orbital contributions^a^
S_1_	465.12	0.0085	H-1 → L (62%)	447.11	0.74	H → L (97%)
S_2_	456.77	0.0014	H-3 → L (51%)	436.78	0.0017	H-1 → L (99%)
S_3_	455.24	0.084	H-2 → L (80%)	430.72	0.00016	H-2 → L (90%)
S_4_	445.90	0.74	H → L (73%)	427.28	0.00031	H-3 → L (90%)
S_5_	379.28	0.057	H-4 → L (90%)	374.81	0.0074	H- 4 → L (91%)

a H: HOMO; L: LUMO. Only the dominating contribution is indicated.

Analyzing the TDDFT excitation energies, we find that for both structures, the dominating transition with the highest oscillator strength occurs at similar wavelengths (445.90 and 447.11 nm) corresponding to the local π–π* excitation of the fluorescein moiety with the HOMO–LUMO character (Figure 3). This excitation gives rise to the main absorption band in the simulated spectra (Figure 4). In addition, the spectra of both structures exhibit a shoulder at 379 and 374 nm (approximately at 425 nm in the shifted spectra; Figure 4), corresponding to another local fluorescein π–π* transition with the HOMO–4 to LUMO character. However, in the case of FlO-ZIF-8, this shoulder is more pronounced than in the case of FlCOO-ZIF-8, thus showing more similarity with the experimental absorption spectrum of F@ZIF-8 (black, dashed line in Figure 4). Based on our experimental results, the λ_max_ of fluorescein in ethanol is at 490 nm with a single absorption peak (Figure S2), while the absorption band shows a red shift to 499 nm with a shoulder around 465 nm for F@ZIF-8 in the solid phase (Figure 4). Thus, the additional shoulder must be associated with interactions between fluorescein and ZIF-8. Comparing the experimental spectrum with the TDDFT results (Figure 4), we assign the shoulder to the HOMO–4 to LUMO π–π* transition located on the fluorescein moiety. The fact that this shoulder is more pronounced in FlO-ZIF-8 than in FlCOO-ZIF-8 indicates that FlO-ZIF-8 may be the more probable interaction geometry, which is consistent with the complexation energies presented above. However, both structures may still coexist. Overall, our computational studies confirm that fluorescein can interact with ZIF-8 by coordinating either its carboxylate or phenoxide group with the open Zn sites on ZIF-8, and both coordinations are thermodynamically equally favorable. However, our predictions also show that the majority of fluorescein may interact with ZIF-8 through the phenoxide-Zn coordination based on the comparison of computed and experimental UV/vis absorption spectra and complexation energies. This also explains why surface adsorption is unavoidable when fluorescein is mixed with preformed ZIF-8 in a wet condition as we show previously in our second control experiment. ZIF-8 has a Zn-rich surface with positive ζ potential in ethanol as confirmed by our previous studies.^77^ Fluorescein can be adsorbed on the ZIF-8 surface through the phenoxide coordination to open Zn sites through postsynthetic modification. Because of the strong surface bonding, it is difficult to wash off surface-adsorbed fluorescein.

**Figure 3:**
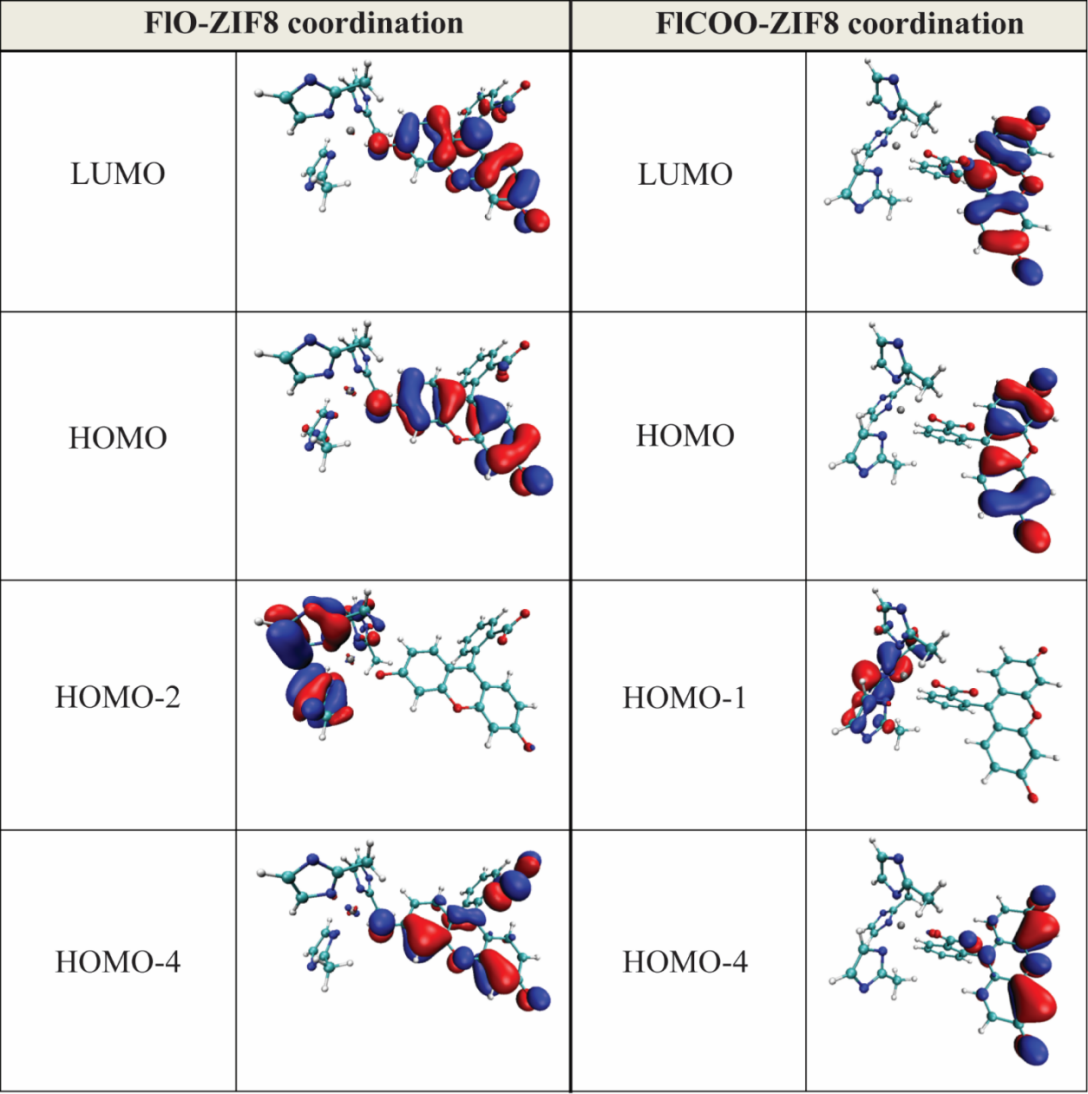
Visualization of the predicted main frontier Kohn–Sham (B3LYP/TZVP) orbitals involved in the three excited states with the highest oscillator strengths for the phenoxide-bound fluorescein with ZIF-8 (FlO-ZIF-8, left) and the carboxylate-bound fluorescein with ZIF-8 (FlCOO-ZIF-8, right) models based on the equilibrium structures optimized by B3LYP/LANL2DZ/D3. Carbon (teal), hydrogen (white), nitrogen (blue), oxygen (red), and zinc (gray); positive and negative lobes are presented by red and blue colors with an isosurface, respectively.

**Figure 4:**
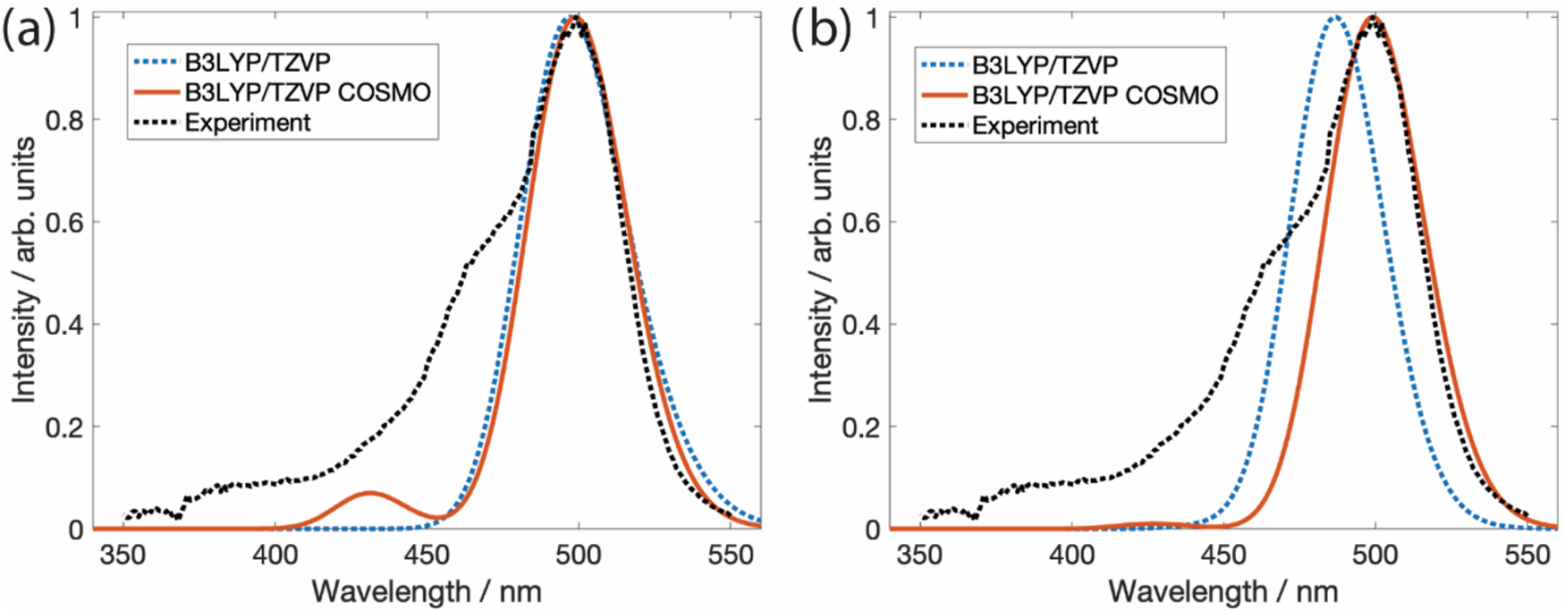
Comparison of experimental and predicted absorption spectra of dianionic fluorescein@ZIF-8 through the phenoxide-bound (FlO-ZIF-8, (a)) and carboxylate-bound configurations (FlCOO-ZIF-8, b). Computed at the TDDFT/B3LYP/TZVP level. COSMO indicates the usage of an implicit solvent mimicking the ethanol solvent. All computed spectra were shifted by +52 nm to match the peak maximum of the experimental absorption spectrum of F@ZIF-8 in ethanol (black dashed).

### Fluorescein Loading Studies

Our next question is to find out whether the loading amount of fluorescein will have an impact on the luminescence properties of F@ZIF-8. Fluorescein is known for its high fluorescence QY (0.79).^78^ In this part, we synthesize F@ZIF-8 with fluorescein doping levels ranging from 0.3 to 30 mol %, and we study their QYs as a function of the fluorescein loading amount (Figure 5). According to our results, a fluorescein level of 0.3 mol % produces the highest QY (69.7 ± 0.1%), whereas a fluorescein level of 30 mol % produces the lowest QY (3.4 ± 0.2%). Interestingly, the pure fluorescein powder shows almost no fluorescence, with a QY of 0.4 ± 0.1%. The QY of F@ZIF-8 decreases with an increase of mol % of fluorescein. This observed quenching is possibly due to the aggregation of fluorescein within ZIF-8 when the loading amount increases.

**Figure 5:**
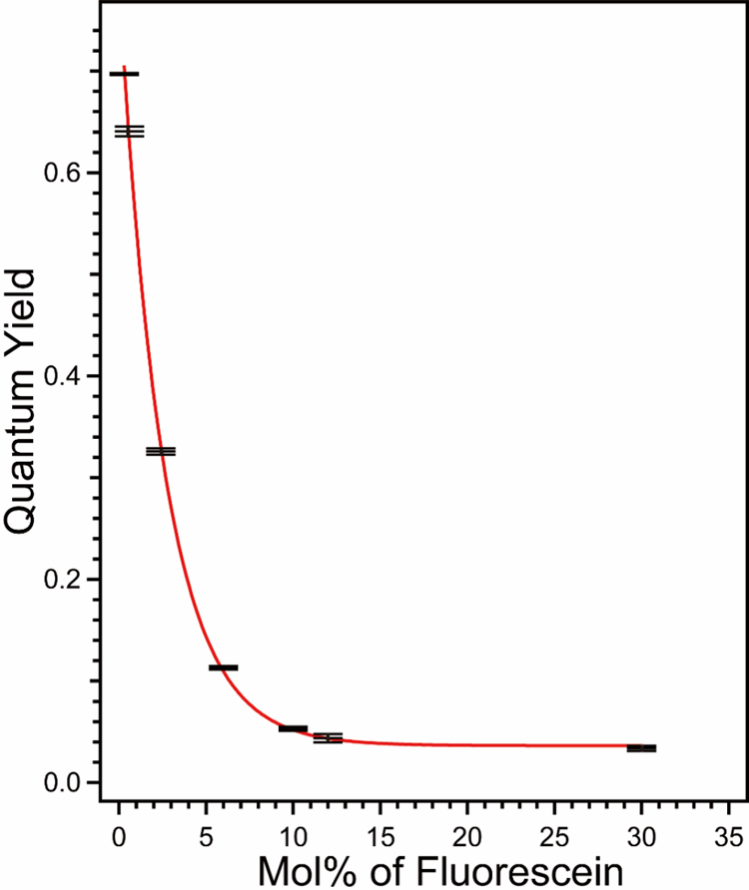
Quantum yield of F@ZIF-8 as a function of fluorescein doping levels. The data were fitted with an exponential function, as indicated by the plot. Error bars indicate standard deviation *N* = 3.

To check the fluorescein loading effect on the ZIF-8 structure, we compared the PXRD patterns of ZIF-8 with variable amounts of fluorescein (Figure 6). No significant change is found in the PXRD patterns, indicating that the crystal structure and overall crystallinity of ZIF-8 remain intact regardless of the fluorescein doping amount. Stokes’ hydrodynamic radius (*R*_h_) of fluorescein is around 5.0 Å,^79,80^ suggesting that fluorescein is too large to diffuse into ZIF-8 through the four-member-ring pore aperture (3.4 Å) in a solvent. However, because the F@ZIF-8 is synthesized in one-pot, fluorescein molecules can be encapsulated within the pores during the crystallization process. The largest sphere that can fit into the ZIF-8 cage has a diameter of 11.60 Å,^45^ which is large enough to accommodate a few fluorescein molecules without introducing too much strain or crystalline defect. These XRD results again confirm our conclusions in the last part that fluorescein molecules can be trapped within ZIF-8 cages. However, when more fluorescein molecules are loaded into ZIF-8, more than one molecule can be trapped inside a single cage, resulting in an aggregation-caused quenching (ACQ) or a fluorescence contact quenching.

**Figure 6:**
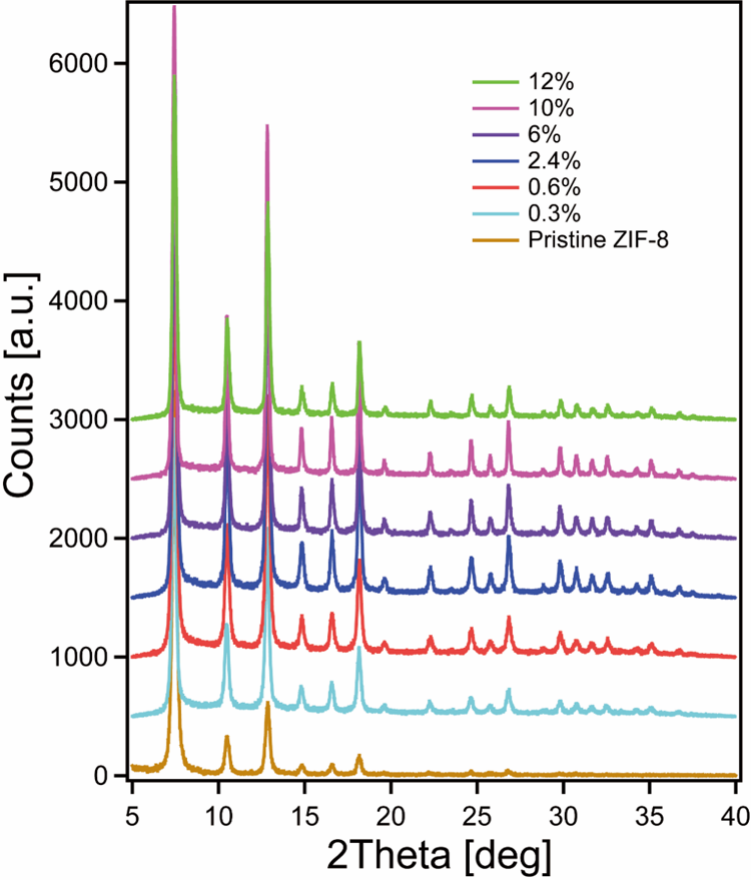
Powder XRD patterns of F@ZIF-8 synthesized with variable fluorescein loading amounts.

While XRD patterns remain invariant, the vibrational spectra of F@ZIF-8 are noticed to be more distinct from that of pristine ZIF-8 when the doping level is greater than 2.4%, as shown in Figure 7. The most obvious changes are found at 1740 and 1220 cm^–1^. Other regions with variations correlate with an increasing loading amount but are convoluted by the vibrational bands of pristine ZIF-8. Markuszewski and Diehl distinguished between the charge-neutral tautomeric solids based on the infrared absorptions in the 1600–1800 cm^–1^ region.^81^ However, the spectrum of a neat solid is influenced by sampling (i.e., ATR crystal, KBr, Nujol, etc.), thereby making assignments by direct comparison unreliable. Therefore, we perform DFT cluster calculations to identify these unique vibrational modes of F@ZIF-8. We compute two models: a fluorescein dianion interacting with a single ZIF-8 cluster with a Zn open site through either a carboxylate or phenoxide group. The simulated frequencies of F@ZIF-8 are computed at the B3LYP/LANL2DZ level (Figure S5). Based on Diehl’s work,^81^ the infrared absorption in the range of 1600–1800 cm^–1^ is related to spiro-lactone carbonyl stretching frequencies, which can be explained by hydrogen bonding with the quinonic xanthene moiety of fluorescein. Based on our DFT vibrational spectral calculations (Figure S5), the peak at 1740 cm^–1^ (marked with an asterisk in Figure S5) corresponds to the mixed symmetric and asymmetric stretching modes of C=C, C=O, and C–O bonds in the xanthene ring. As a result, fluorescein is completely localized, which explains the lack of overlapping frequencies from the ionized cation from ZIF-8. The quinone oxygen interactions have a strong influence on the tautomeric equilibrium. As the character of the quinone increases, the carboxylate gradually approaches the carboxylic acid. Conversely, as the quinone oxygen becomes more sp^3^-hybridized, the carboxylate gradually approaches the spiro-lactone. The ZIF-8-related bands in the region 1300–1600 cm^–1^ in general appear to exhibit some degree of intensity enhancement as fluorescein loading is increased. Though discrete assignment is difficult, these bands are mostly attributable to various aryl C–H wagging modes with contributions from both fluorescein and imidazolate. A triplet at 1240–1220 cm^–1^ is observed with perpendicular in-plane stretching frequencies from both xanthene and pendant benzoate, indicating that there may be some minor interaction between Zn^2+^ and carboxylate, but deconvolution is difficult. Overall, the increased doping level allows for detectable signals in the vibrational spectra, enabling a better understanding of the interactions between fluorescein and ZIF-8.

**Figure 7:**
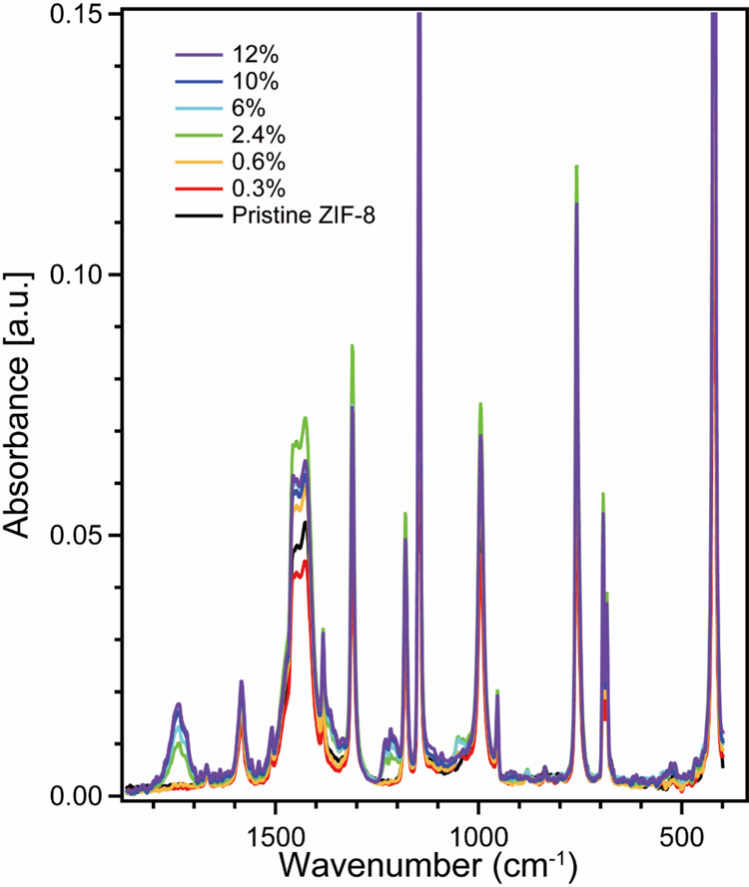
ATR-IR spectra of F@ZIF-8 synthesized with variable fluorescein loading amounts.

### Detection of Explosives

Nitroaromatics are effective fluorescence quenchers as the nitro group is an electron-withdrawing group, where photoinduced electron transfer (PET) can occur when they are in contact with fluorophores, which are electron-rich molecules or conjugations. In our case, fluorescein is loaded within ZIF-8 through phenoxide bonding and results in excellent fluorescence through host–guest interactions. We can utilize the fluorescence behavior of F@ZIF-8 to detect explosives. We investigate the fluorescence quenching response of F@ZIF-8 to six NAC analytes at various concentrations, summarized in Table S4. Figure 8 shows the quenching percentages of F@ZIF-8 after exposure to 200 μL of different explosive analytes at a concentration of approximately 0.1 wt %. The fluorescence emission intensity is normalized to the F@ZIF-8 solution without exposure to any explosive. Notably, F@ZIF-8 exhibits a strong “turn-off” fluorescence response to TNP, with nearly 97 and 45% quenching upon exposure to 200 μL of ∼1 and ∼0.1 wt % TNP solution, respectively. Our F@ZIF-8 material also demonstrates detectable responses to TNT and NT, as well as to pure NB and NP. However, fluorescence quenching experiments with 1 mg/mL of RDX showed a negligible quenching effect. The quenching percentage appears to correlate with the analyte concentration: 1% TNP, pure NB, and NP induce more significant quenching than the lower-concentration exposures.

**Figure 8:**
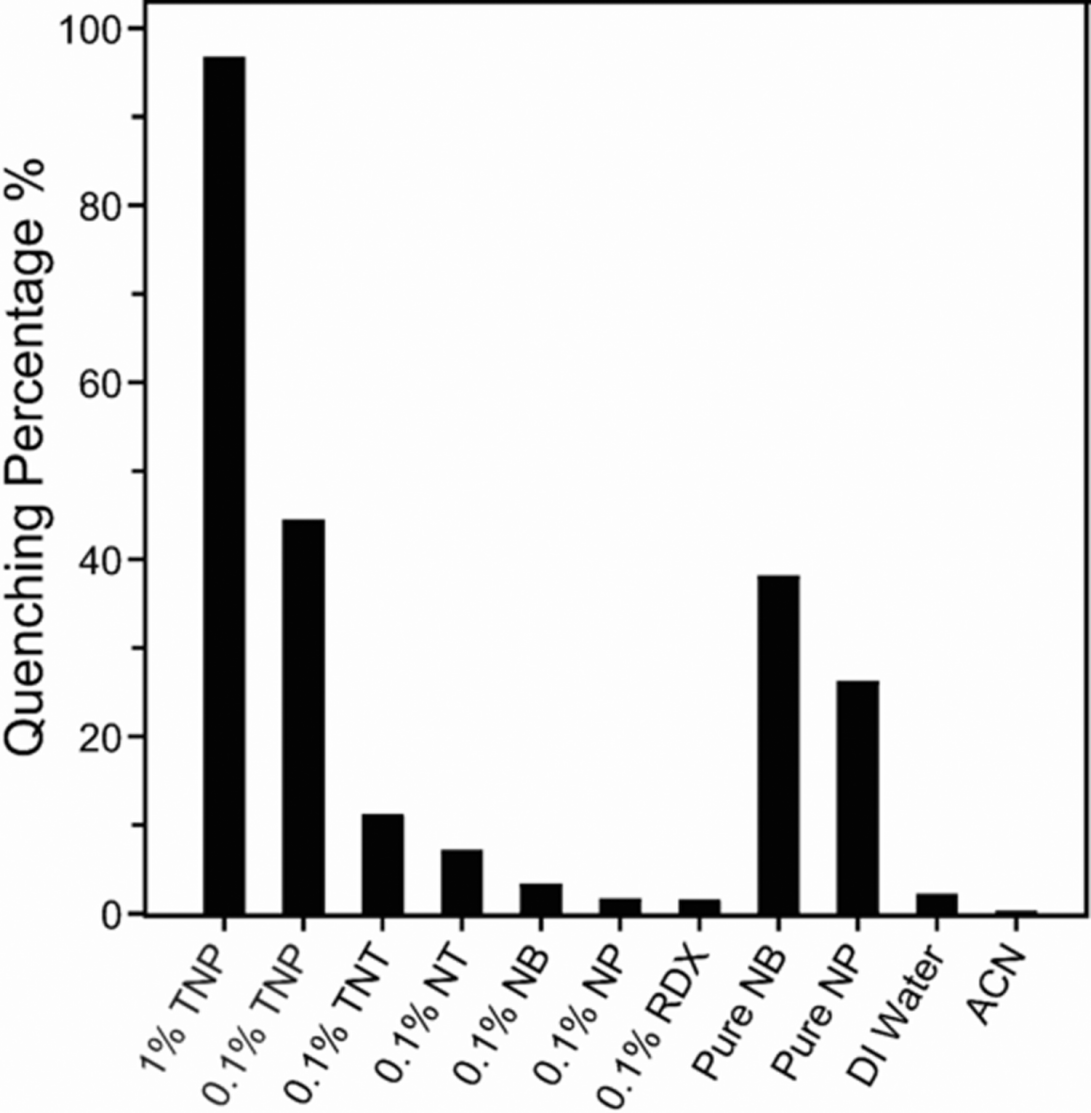
Fluorescence quenching percentages of F@ZIF-8 by various explosive analytes and two solvents—deionized (DI) water and acetonitrile (ACN)—at room temperature. The F@ZIF-8 solution has a concentration of 0.4 mg/mL in ACN. The tested explosives are compared with a concentration of approximately 0.1 wt % in a solvent; the detailed concentrations are listed in Table S4.

To further study the concentration-dependent sensing behavior of F@ZIF-8 toward TNP, we monitored its fluorescence quenching upon the incremental addition of TNP. Figure 9a shows a series of representative emission spectra of F@ZIF-8 solution after successive additions of 20 μL of TNP (∼1 wt %), up to a total volume of 200 μL. The fluorescence quenching of F@ZIF-8 increases progressively with the amount of TNP added. We also quantify the quenching percentage as a function of the cumulative volume of TNP added to the F@ZIF-8 solution (Figure 9b). All fluorescence emission intensities are normalized to the emission of F@ZIF-8 prior to TNP addition. The initial quenching caused by 20 uL of TNP (∼1 wt %) is already substantial, 43.0 ± 6.7%—compared to the responses to other explosives shown in Figure 8, indicating the high sensitivity of F@ZIF-8 toward TNP. With the continued addition of TNP, the quenching of F@ZIF-8 increases, reaching 97.2 ± 1.1% after the addition of 200 uL of TNP (Figure 9b). The quenching efficiency of TNP to F@ZIF-8 is analyzed by using the Stern–Volmer (SV) equation (eq 1), as shown in Figure 9c. At TNP concentrations above 2 mM, the SV plot deviates from linearity, which may be attributed to a combination of dynamic (collisional) and static quenching, as well as diffusion limitations from the MOF host.^82–84^

**Figure 9:**
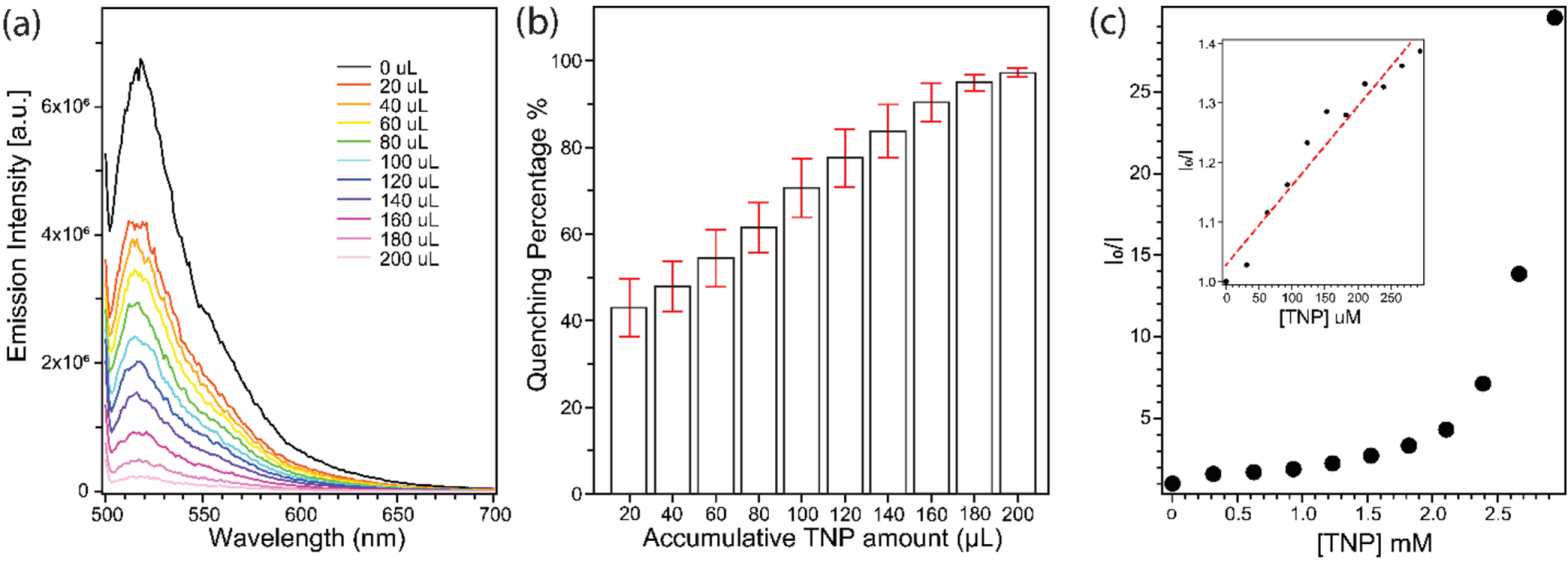
Fluorescence emission spectra (a) and the corresponding fluorescence quenching percentage (b) of F@ZIF-8 dispersed in acetonitrile after exposure to the incremental amount of a TNP solution (∼1 wt %); and (c) the corresponding Stern–Volmer plots of F@ZIF-8 with TNP as a quencher at different concentrations; the plot of lower concentrations is shown in the inset. Error bars indicate standard deviation, *N* = 3.

We further calculate the limit of detection (LOD) based on eq 2. The LOD determination relies on the corresponding SV plot; as only concentrations within the dynamic linear range can be used for accurate calculation. The LOD for TNP is found to be 2 μM (equivalent to 0.46 mg/L), which can be used for detecting the permissible level for TNP in drinking water (0.5 mg/L).^85^ The determined LOD for TNP is comparable to values reported from other LMOFs.^86–89^ The LOD values for other explosives tested in this study are listed in Table S5. Overall, F@ZIF-8 exhibits excellent sensitivity toward TNP. More importantly, compared to other reported sensors, F@ZIF-8 is easy to prepare and can be adapted into strips in the future for fast detection.

## Conclusions

In summary, we report a one-pot synthesis method to prepare luminescent MOF nanoparticles by doping ZIF-8 with fluorescein under ambient conditions. The resulting F@ZIF-8 nanocrystals exhibit strong fluorescence with a QY of up to ∼70%. Our combined spectroscopic, X-ray diffraction, and gas sorption studies confirmed that most fluorescein molecules are trapped inside of ZIF-8 cages, but we cannot rule out the possibility that some fluorescein may be adsorbed on the outer surface of ZIF-8 as both locations contribute to its fluorescent behaviors. Our computational studies confirmed that the interactions between fluorescein and ZIF-8 are through coordination between the deprotonated phenoxide from fluorescein and the open metal sites from ZIF-8. Due to the limited pore volume in each cage of ZIF-8, fluorescein can be separated from each other, thus enhancing its overall QY. We notice that the QY of F@ZIF-8 decreases with an increase of the fluorescein doping amount. This confirms that the framework of ZIF-8 prevents the ACQ effect by effectively separating fluorescein molecules when the loading amount is low. We also demonstrate the potential of using these F@ZIF-8 nanoparticles for the detection of nitroaromatic explosives. The F@ZIF-8 shows a “turn-off” fluorescence response for several nitroaromatic analytes, especially for detecting TNP. Our material shows great potential to engineer a compact portable spectrofluorometer using prepacked glass capillaries as an on-the-go detection device, thus promoting the design of molecular explosive devices with high sensitivity, excellent selectivity, and fast responsivity. This work also provides insight into other fluorescent material designs for explosive detections.

## Supplementary Material



## Notes

Chemicals, synthesis details, UV–vis spectra, fluorescence emission spectra, DFT calculation results, explosive testing, and additional SEM images (PDF)
