# Supplementary material for: Fluorescent Metal–Organic Framework Nanoparticles for Explosive Detection
Source: J Phys Chem C Nanomater Interfaces. 2025 Jun 5;129(24):10866–78. doi: 10.1021/acs.jpcc.5c00753 (PMC12186613; doi:10.1021/acs.jpcc.5c00753)
Supplement: Supplementary file 1 [file jp5c00753_si_001.pdf]

Fluorescent Metal-Organic Framework Nanoparticles for Explosive Detection

Raymond Yu[1], Tiffany Nguyen[1], Victor H. Cortez[1], Boyang Chen[1], Kristi M. Ishihara[1], Enrico Tapavicza[1,2] and Fangyuan Tian[1]**

1. Department of Chemistry and Biochemistry, California State University Long Beach, Long Beach, California USA 90840

2. Faculty of Chemistry and Pharmacy, Institute of Physical and Theoretical Chemistry, University of Regensburg, 93040 Regensburg, Germany

Supporting Information

Table of Contents

1. Chemicals and synthesis conditions……………………….……….…………………………*S-2*

2. Control experiments……………….………………….……….…………………………*S-2*

3. Additional material characterization……………...……………….…………………….*S-3*

4. Additional computational results………………..………………….…………………….*S-5*

5. Explosive detection…..…..……………………..……………….……….……………….*S-7*

6. Metathesis details………..…..…………………..……………………..………………..…*S-11*

1. Chemicals and Materials.

All chemicals were reagent grade or better, used as received, and included zinc nitrate hexahydrate ($Zn(NO_3)_2 \cdot 6H_2O$, Fisher Chemical, ACS Certified), 2-methylimidazole (2-mIm, Acros, 99%), fluorescein (Fisher Chemical, 90+%), uranine (Concentrated, Water Soluble/Laboratory, Fisher Chemical), methanol (Fisher Chemical, ACS Grade), and ethanol (Thermo Scientific, 200 Proof). The chemical details of the tested explosive analytes are discussed in session S4.

2. Control experiments of mixing fluorescein and pre-formed ZIF-8

Control experiment #1: Mix fluorescein powder and pre-formed ZIF-8 solid in a molar ratio of 0.3 : 100.

Control experiment #2: Add fluorescein (0.3 mol%) into the ZIF-8 synthesis solution after the last cycle of centrifugation. Then, the ethanol solvent was removed using rotavap and the collected powder was dried in a vacuum oven at 90 ºC overnight.

Control experiment #3: Add fluorescein (0.3 mol%) into the ZIF-synthesis solution after the last cycle of centrifugation. The mixture was further rinsed with ethanol for another three times. After that, the collected powder was dry in a vacuum oven at 90 ºC overnight.

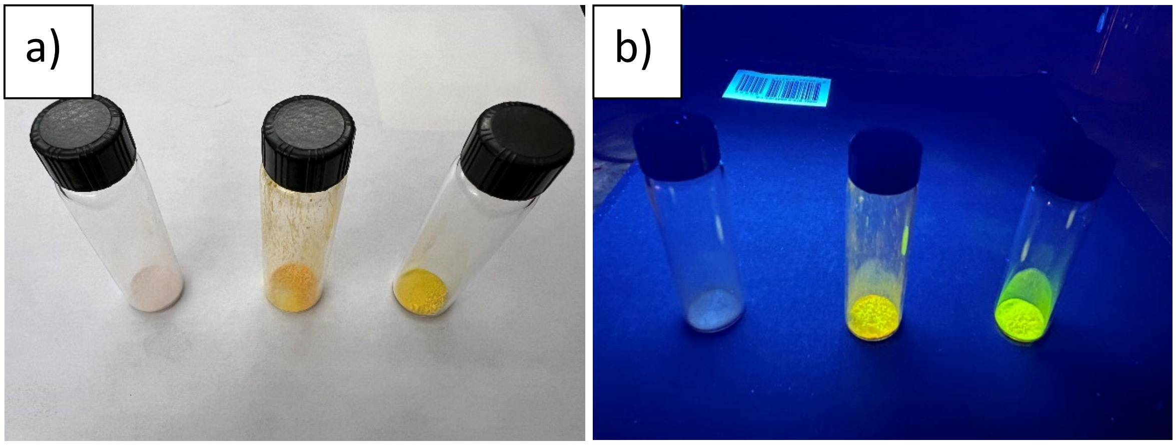

Figure S1. Optical images of a mixture of pristine ZIF-8 with solid fluorescein (control experiment #1, left), ZIF-8 with fluorescein added after the third centrifuge (control experiment #2, middle) and after another three-time rinse in ethanol (control experiment #3, right) under visible light (a) and UV lamp (b).

2. Additional material characterization results

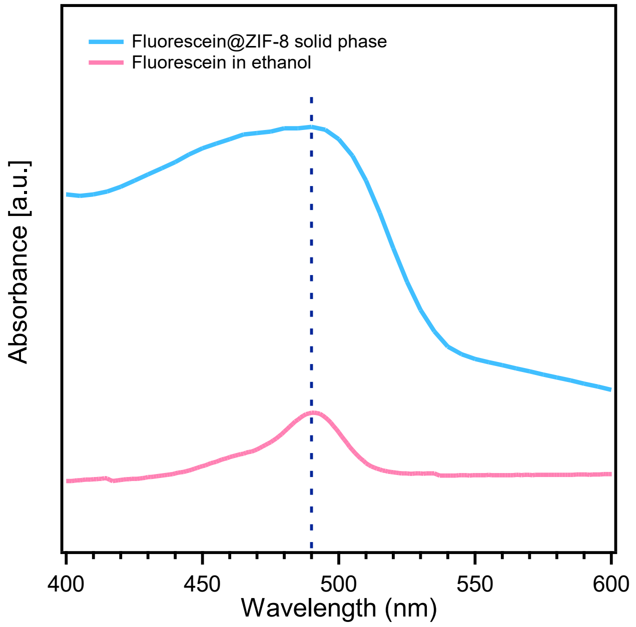

Figure S2. Absorption spectra of fluorescein in ethanol and solid-state fluorescein@ZIF-8.

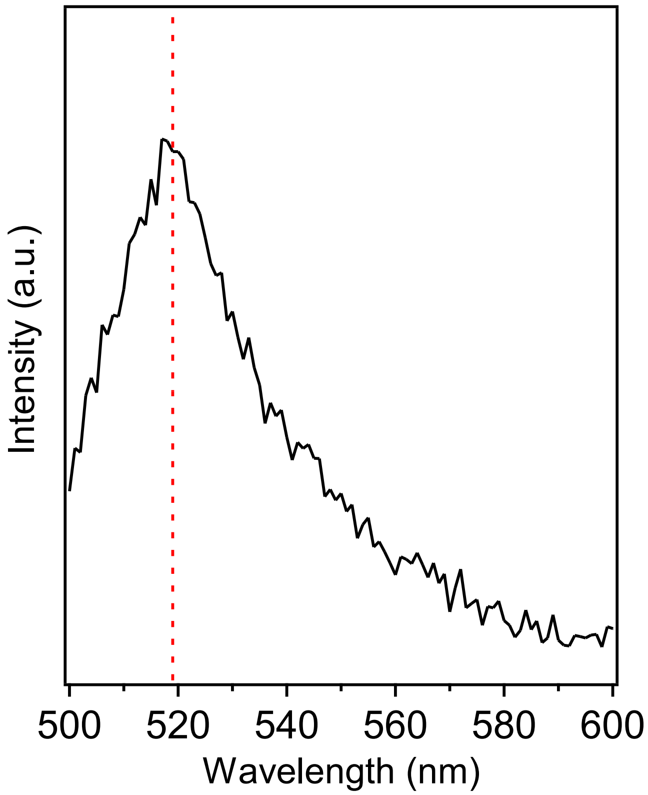

Figure S3. Emission spectrum of solid-state fluorescein@ZIF-8. Excitation wavelength was set at 490 nm. $\lambda_{emission}$ = 519 nm.

Table S1. List of porosity and surface areas computed from gas sorption measurements

Doping level	BET surface area (m^2 /g)	t-Plot micropore area (m^2 /g)	t-Plot external surface area (m^2 /g)	t-Plot micropore volume (cm^3 /g)
**0%**	1738.8614	1603.3564	135.5050	0.640698
**0.3%**	1734.4929	1641.5437	92.9492	0.648526
**0.6%**	1707.1584	1607.0753	100.0830	0.633124
**2.4%**	1721.3188	1660.1951	61.1238	0.660078
**3.0%**	1695.3250	1636.1834	59.1416	0.648939
**6.0%**	1563.8315	1530.7890	33.0425	0.605722
**10.0%**	1495.0399	1483.5651	11.4748	0.563383

4. Additional computational results

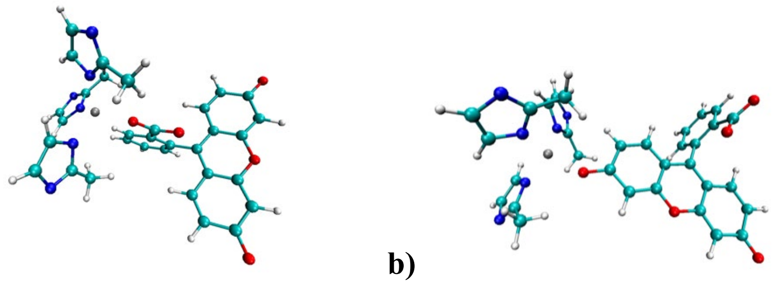

a) b)

Figure S4. Ground state equilibrium structures of a) carboxylate-bound fluorescein with ZIF-8 (FlCOO-ZIF8) and b) phenoxide-bound fluorescein with ZIF-8 (FlO-ZIF8). Computed at the B3LYP/LANL2DZ level with Grimme D3-dispersion correction.

Table S2. List of computed total energies, basis set superposition error (BSSE), and complexation energies* of carboxylate-bound (FlCOO-ZIF8) and phenoxide-bound fluorescein with ZIF-8 (FlO-ZIF8).

	**Counterpoise corrected energy (au)**	**BSSE energy (au)**	**sum of fragments (au)**	**Complexation energy (raw) (kcal/mol)**	**Complexation energy (corrected) (kcal/mol)**
**FlO-ZIF8 geometry**					
	-2004.52579206	0.009937684181	-2004.56563707	18.77	25.00
D3-correction	-2004.60957032	0.010275727810	-2004.63976058	12.50	18.94
**FlCOO-ZIF8 geometry**					
	-2004.52378649	0.010795405427	-2004.56431835	18.66	25.43
D3-correction	-2004.61005741	0.011874130485	-2004.63706295	9.50	16.95

* Counterpoise corrected calculations of the interaction energies were performed at the B3LYP/LANL2DZ level with and without Grimme D3-dispersion correction.

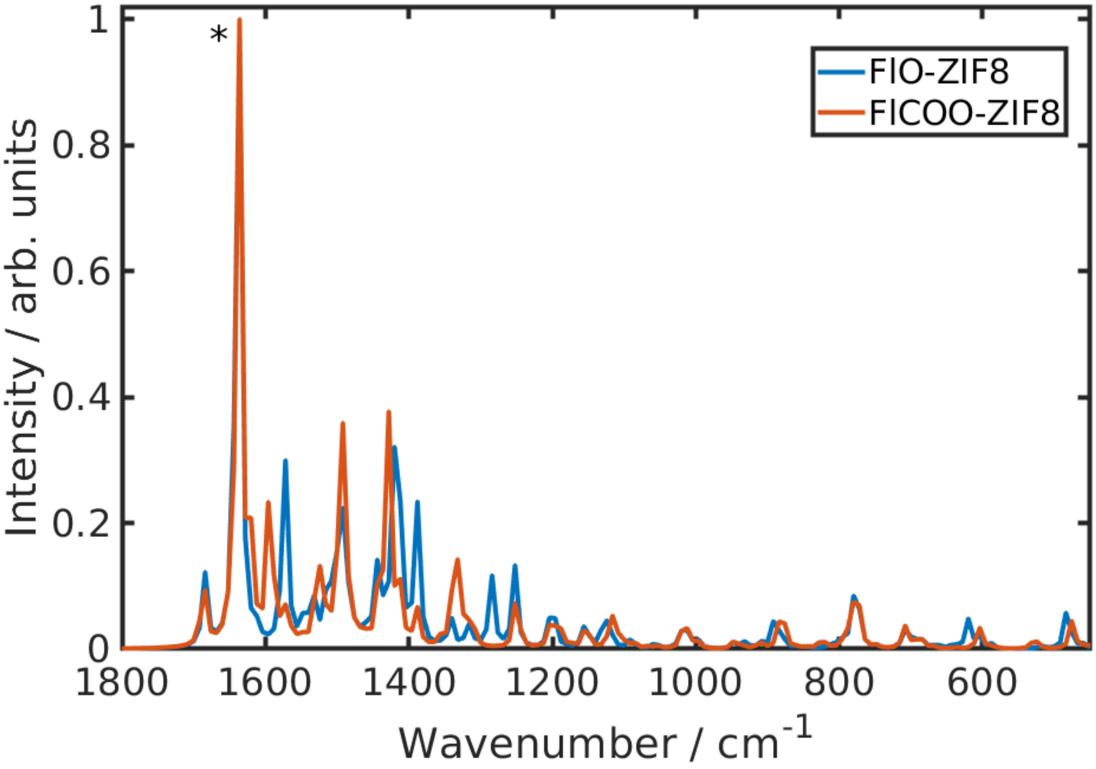

Figure S5. Simulated vibrational spectra of the phenoxide-bound fluorescein ZIF-8 (FlO-ZIF8, blue) and the carboxylate-bound fluorescein ZIF-8 (FlCOO-ZIF8, red). Frequencies computed at B3LYP/LAN2LDZ level. A Lorentzian broadening function was applied with FWHM at 10 cm^{-1}.

Table S3. CC2/SVP excitation wavelengths and oscillator strengths for the lowest five excited states.

	FlO-ZIF8		FlCOO-ZIF8	
	**Wavelength (nm)**	**Oscillator strength (au)**	**Wavelength (nm)**	**Oscillator strength (au)**
**S_1**	468.57	0.88999205	466.64	0.81107276
**S_2**	421.68	0.15302299	393.09	0.00007530
**S_3**	403.24	0.00007462	384.15	0.00000996
**S_4**	368.57	0.02007226	350.33	0.04548301
**S_5**	355.97	0.01856006	309.95	0.02513387

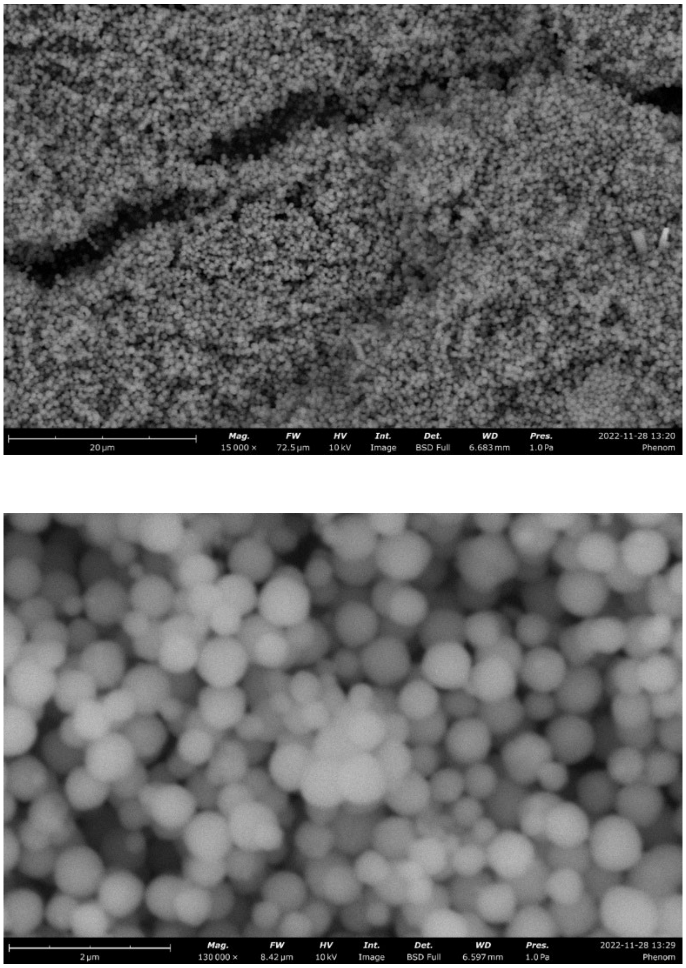

Figure S6. SEM images of 12 mol% F@ZIF-8

5. Explosive detection

Unless otherwise stated, fluorescence measurements were conducted using a solution of 1 mg F@ZIF8 to 2.5 mL of ACN. The solution was sonicated until all large particles were dispersed into a cloudy suspension and tested within the same day. All spectra were taken on a Shimadzu RF-6000 with an excitation wavelength of 490 nm and recorded from 500 - 700 nm.

Explosive Compound Quenching: Six nitroaromatic compounds were tested for their quenching ability, Table S4. A cuvette was filled with 2.5 mL of F@ZIF-8 solution and set to stir in the fluorometer. After an initial reading, 20 µL of analyte were added and another measurement was taken. This process was repeated until a total of 200 µL total were added.

Table S4. A list of tested explosive analytes in this study.

Abbreviation	Chemical Name	Molecular Structure	Concentration (mg/mL)	Solvent	Source
TNP	2,4,6-Trinitrophenol	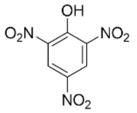	9.1	DI water	Fisher Scientific, SP920050, used as it or after dilution.
			0.91	acetonitrile	
TNT	Trinitrotoluene	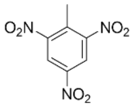	1	acetonitrile	Millipore-Sigma, ERT-022S, used as it.
NT	4-Nitrotoluene	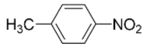	1	acetonitrile	Thermo Scientific, 129051000, diluted in our lab.
NB	Nitrobenzene	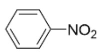	1	acetonitrile	Acros, A0402201, used as it or after dilution.
			Purity 99+%	N/A	
NP	2-Nitropropane	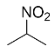	1	acetonitrile	Millipore-Sigma, 130265, used as it or after dilution.
			Purity 96+%	N/A	
RDX	1,3,5-trinitro-1,3,5-triazinane	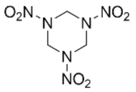	1	acetonitrile	Millipore-Sigma, ERR-001S, used as it.

Reusability Trials: A master solution of F@ZIF8 was made and split into three 15 mL centrifuge tubes with 6 mL in each. Fluorescence emission spectra were collected before and after adding 20 uL of ~1 wt% TNP. After each run, tubes were centrifuged for 5 min at 7500 rpm. Next, 6 mL of fresh ACN were added to each test tube, and the test tubes were shaken to help with dispersion.

This process was repeated until a total of five cycles were completed and all the samples were tested subsequently.

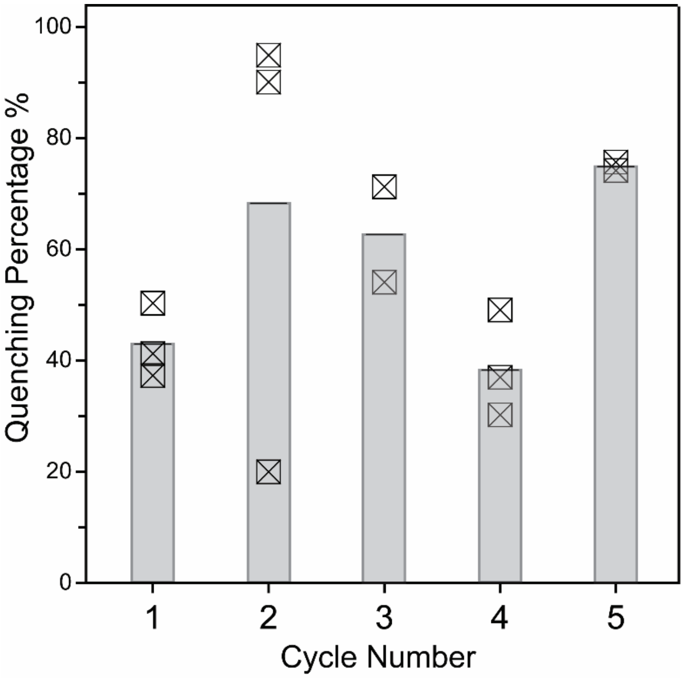

Figure S7. The quenching percentage of F@ZIF-8 toward 20 uL of TNP (0.91 wt%) for 5 cycles.

Long Term Leeching Trials: To test the leeching of the fluorescein from ZIF-8 host, a 8-day leaching study was done with Slide-A-Lyzer™ MINI Dialysis Devices from Thermo Fisher. 2 mL of a 5 mg/ mL solution of F@ZIF8 in ACN were placed in a well of a 50 mL dialysis tube. The tube was filled with 43 mL of ACN and left to lightly shake at 170 rpm at room temperature. After a 24 h period, a 3 mL sample was collected from the tube, 3 mL of fresh ACN were added, and the tube was returned to the shaker. Samples were collected every day for seven days except for one day when an extra 1 mL of solvent was added due to insufficient contact with the well. All samples were tested together after the trial period.

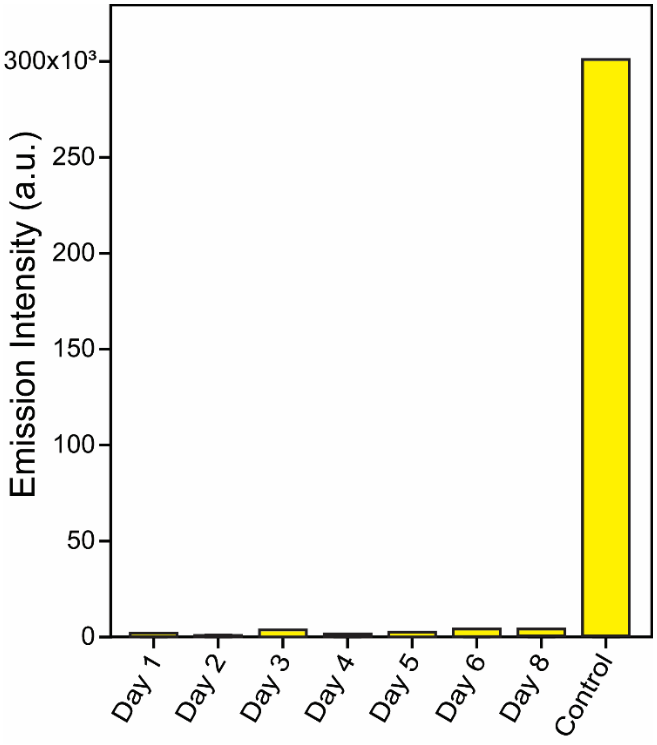

Figure S8. Leaching test of F@ZIF-8 in acetonitrile for 8 days compared with a control of the same concentration of F@ZIF-8 fully released in acetonitrile.

Table S5. A summary of LOD toward various explosives by F@ZIF-8 in this study

Analyte	***LOD***
TNP	2 uM
TNT	30 uM
NT	191 uM
NB	51 mM
NP	72 mM
RDX	N/A*

* The linear correlation is poor at the tested low concentration conditions, thus no LOD can be calculated.

Metathesis Reaction Details

The order of operations in the synthetic procedure (of F@ZIF-8) calls for the dissolution of the dye in the 2-mIm precursor solution before combining with the Zn^{2+} precursor solution. In one synthesis experiment two variables were changed. The Zn^{2+} precursor solution was doped first with the uranine, the disodium salt of fluorescein (Na_2Fls). Surprisingly, it was discovered that these conditions lead to the precipitation of a reddish orange product. The solid was isolated by filtration and the material gave no XRD signal, indicative of an amorphous phase. The vibrational spectrum (Fig. S9), however, had characteristics of both fluorescein and uranine. The crude was thus tentatively identified as a product of the salt metathesis reaction.

$$Zn(NO_3)_2 + Na_2Fls \longrightarrow 2\,NaNO_3 + ZnFls$$

This discovery revealed a strong electrostatic interaction between Zn^{2+} and anionic fluorescein. The implication is that the kinetics behind F@ZIF-8 nucleation is probably quite complex; necessarily involving competitive coordination of Zn^{2+} by 2-mIm and fluorescein. Apparently both organic components can act as Lewis Bases but must conceivably act as Bronsted acid/base pairs in the mildly alkaline conditions of the original synthetic procedure. While no formal kinetics study was performed, it was qualitatively observed that increased fluorescein concentration does indeed reduce the amount of F@ZIF-8 that can be obtained in the one-hour reaction time. While the metathesis reaction revealed greater complexity in the kinetics of F@ZIF-8 synthesis, the product itself is of reduced complexity as it is a binary system. This provided a good starting point for *ab initio* computational experiments.

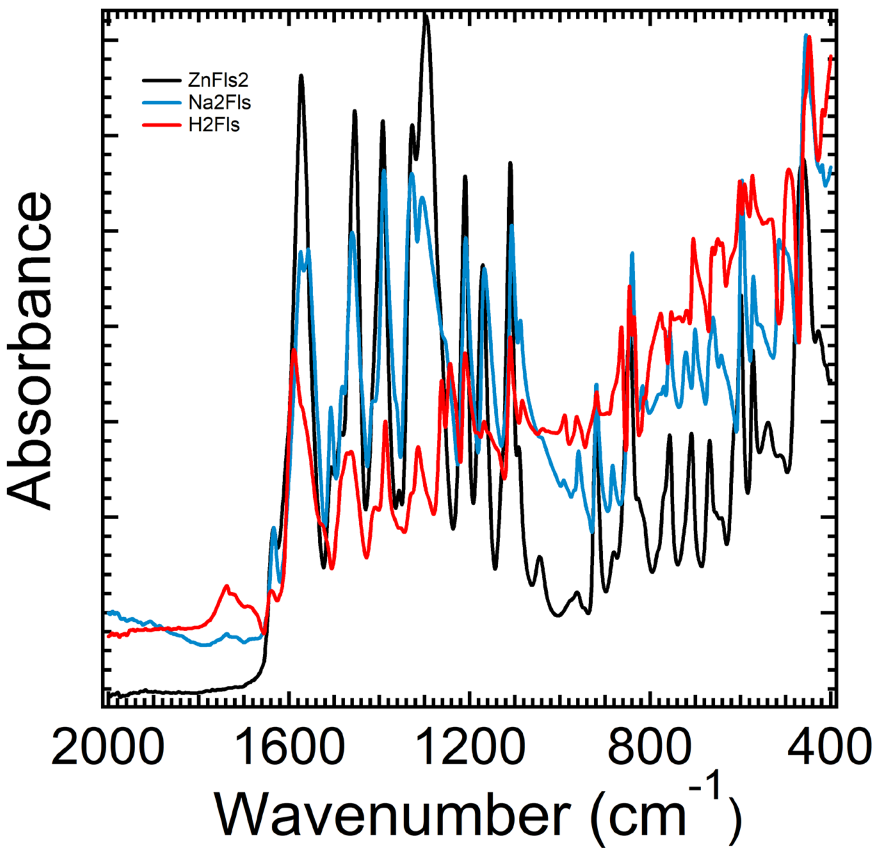

Figure S9. Experimental vibrational spectra of fluorescein and sodium and zinc salts.
